# Alzheimer's mice model APP‐knock‐in displays altered hippocampal cholinergic function

**DOI:** 10.1002/alz70855_106449

**Published:** 2025-12-24

**Authors:** Sumonto Mitra, Ruchi Gera, Simone Tambaro, Per Nilsson, Bengt Linderoth, Homira Behbahani, Taher Darreh‐Shori, Maria Eriksdotter

**Affiliations:** ^1^ Division of Clinical Geriatrics, Dept.of NVS, Karolinska Institutet, Huddinge, Stockholm, Sweden; ^2^ Division of Neurogeriatrics, Dept of NVS, Karolinska Institutet, Solna, Stockholm, Sweden; ^3^ Department of Clinical Neuroscience, Karolinska Institutet, Stockholm, Stockholm, Sweden; ^4^ Division of Clinical Geriatrics, NVS department, Karolinska Institutet, Huddinge, Stockholm, Sweden; ^5^ Center for Alzheimer Research, Division of Clinical Geriatrics, Department of Neurobiology, Care Sciences and Society, Karolinska Institutet, Huddinge, Sweden; ^6^ Theme Inflammation and Aging, Karolinska University Hospital, Huddinge, Stockholm, Sweden

## Abstract

**Background:**

The hippocampal region of the brain is a crucial site of neurotrophic factor production like nerve growth factor (NGF) – the master regulator of cholinergic pathways and brain derived neurotrophic factor (BDNF). Hippocampus coordinates memory and cognition and is found affected with amyloid‐beta (Aβ) pathology in AD. Altered cholinergic pathways and memory dysfunction are well‐known changes during Alzheimer's disease (AD). Til date, a clear connection between Aβ pathology in modulating hippocampal cholinergic pathways has not been proven.

**Method:**

Humanized APP‐knock‐in mouse model of AD (*App^NL‐G‐F^*) was utilized, and hippocampus tissue was isolated at 2‐month age (pre‐plaque stage), 7‐month age (plaques present + initiation of cognitive deficits), and 12‐month age (advanced amyloid pathology), and from age‐matched wildtype control mice (C57BL/6JRj) respectively. Total protein was isolated by extracting hippocampal samples in various buffers (soluble, ionic, and detergent soluble fractions) and pooled together. Enzyme assays for acetylcholinesterase (AChE), butyrylcholinesterase (BuChE) and choline acetyltransferase (ChAT) were performed, along with ELISA for the estimation of total NGF and BDNF levels, respectively.

**Result:**

Age‐dependent changes were observed in *App^NL‐G‐F^* mice compared to wild‐type controls. Cholinesterase activity in the *App^NL‐G‐F^* mice was increased for AChE in 7 months, while BuChE activity was higher during all time points, with respect to wildtype control mice. ChAT levels were nominally altered while the cholinergic index (ChAT/AChE) was significantly increased at 2‐months in *App^NL‐G‐F^* mice. NGF levels remained unchanged, but BDNF levels increased in an age‐dependent manner in *App^NL‐G‐F^* mice as compared to control mice.

**Conclusion:**

Significant age‐dependent alterations in cholinergic activity and neurotrophic factors were evident in hippocampus tissue of *App^NL‐G‐F^* mice, when compared to wild‐type counterparts, underlining the importance of the cholinergic activity in relation to amyloid pathology.

